# Effects of microbial inoculants on the fermentation characteristics and microbial communities of sweet sorghum bagasse silage

**DOI:** 10.1038/s41598-020-57628-0

**Published:** 2020-01-21

**Authors:** Miaoyin Dong, Qiaoqiao Li, Fuqiang Xu, Shuyang Wang, Jihong Chen, Wenjian Li

**Affiliations:** 1 Institute of Modern Physics, Chinese Academy of Sciences, 509 Nanchang Rd., Lanzhou, Gansu 730000 P.R. China; 2 Institute of Biology, Gansu Academy of Sciences, 197 dingxi South Rd., Lanzhou, Gansu 730000 P.R. China; 3 College of Life Sciences, University of Chinese Academy of Sciences, No. 19A Yuquan Road, Beijing, 100049 P.R. China

**Keywords:** Applied microbiology, Food microbiology, Microbial ecology

## Abstract

Sweet sorghum bagasse (SSB) is a promising raw material for silage fermentation due to its high residual nutritive, but the efficient fermentation strategy of SSB has not been reported yet. This study evaluated the effects of microbial inoculant on the fermentation quality, chemical composition and microbial community of SSB silage. The silage inoculated with isolated lactic acid bacteria (LpE) achieved better fermentation than that of commercial inoculant A, B (CIA, CIB) and untreatment, including low pH value, high levels of lactic acid and water soluble carbohydrates (WSC) content, which demonstrated that the LpE inoculant could contribute to the preservation of nutrition and the manipulation of fermentation process of SSB. In addition, the results of microbial community analysis indicated that the LpE inoculant significantly changed the composition and diversity of bacteria in SSB silage. After ensiling, the LpE inoculated silage were dominated by *Lactobacillus*(95.71%), *Weissella*(0.19%). These results were of great guiding significance aiming for high-quality silage production using SSB materials on the basis of target-based regulation methods.

## Introduction

Sweet sorghum (*Sorghum bicolor* (L.) Moench) is a low-cost non-food energy crop that can simultaneously produce the sugar juice and bagasse^1^. In addition, the sweet sorghum has a high photosynthetic efficiency resulted in high biomass productivity and rapid accumulation of sugars in stems^2,3^. It also has a remarkable stress tolerance in harsh growth conditions, such as salinity, alkalinity and drought areas^4,5^.

Currently, the utilization of sweet sorghum mainly focused on the juice fermentation for biofuels and chemicals production because the juice extracted from sweet sorghum stems contains high sugar contents and could be fermented directly by microbes^6,7^. Hence, a large amount of sweet sorghum bagasse (SSB) will be certainly left from the industrial-scale extraction of sugar juice to meet the needs of fermentation industry. Although the SSB was used as a substrate for burning, converting to cellulosic sugars, producing biofuels and structural materials^8–10^, it remains underutilized at current stage. While ensiling is an efficient approach to fermenting sweet sorghum bagasse and used for animal feeds^11^. During ensiling process, the lactic acid bacteria (LAB) and water soluble carbohydrate (WSC) are important factors for achieving high quality silage fermentation^12,13^. Thus, the production of high quality silage from sweet sorghum bagasse is a good choice due to its high residual nutritive and sugars, and low cost^10^. The LAB inoculant have been proposed as an effective additives to prolong the storage time and improve the feed palatability^14,15^. The present study also uses two LAB strains (NCBI Accession No. MN022576 and MN022577) as additive, isolated from corn silage. Besides, the application of LAB isolated from forage crops for SSB silage fermentation has not been reported.

In general, silage fermentation is a fully microbial-based fermentation process^16^, thus the assessment of microbial community is necessary to improve our knowledge and understanding about the role of microorganisms in ensiling process. Microbial community of silage was originally studied using classical microbiological or molecular techniques, such as plate count of culture-based, denaturing gradient gel electrophoresis (DGGE) by PCR-amplified^17^. Although these studies could provide a information on the microbial community of silage, its information were narrowed on the species abundance involved in the fermentation process^18^. Therefore, the next generation sequencing (NGS) technology was applied in the study to characterize the microbial communities associated with the SSB fermentation^19,20^.

Thus far, relatively few studies have reported the use of NGS method to explore the microbial community in SSB silage inoculated with LAB additive. Most of previous studies only focused on the utilization potential of SSB as animal feed by natural fermentation^21,22^, but not characterized the changes of microbial community and its ultimate effects on SSB silage quality, which might provide the significant information for manual regulation of SSB fermentation. Therefore, the objectives of this study were to enhance the fermentation quality of SSB by using various microbial inoculants, including commercial corn inoculants and isolated LAB inoculant, and evaluate the effect of various inoculants on nutritive values, fermentation characteristics and microbial community of SSB fermentation. In addition, the evaluation of microbial inoculants on the fermentation quality and microbial community of SSB silage were also firstly reported.

## Results and Discussion

### Characteristics of SSB prior to ensiling

The analysis of chemical composition and microbial counts by plate culture of SSB raw materials were performed and shown in Table 1. In the present study, the DM contents of SSB were 37.3%, which were similar to previous study (38.3%, harvested in October)^10^. The WSC contents of SSB were 171.6 g/kg of DM, which indicated that the SSB forage could achieve good fermentation during ensiling^23^. The contents of cellulose and hemicellulose in SSB were 42.82% and 24.46%, respectively. The contents of NDF and ADF in SSB were 803.23 g/kg and 558.66 g/kg of DM, which were lower compared with the results reported by dos Passos Bernardes *et al*.^21^. Nevertheless, the ensiling process of SSB with high NDF and WSC contents still need to be manipulated by cellulolytic and microbial additives.

**Table 1:** Chemical composition and microbial counts of SSB prior to ensiling.

Items^a^	Sweet sorghum bagasse (SSB)
DM (%, FM)	37.3
pH	5.14
WSC (g/kg, DM)	171.6
NDF (g/kg, DM)	803.23
ADF (g/kg, DM)	558.66
Hemicellulose (%)	24.46
Lignin (%)	13.05
Cellulose (%)	42.82
LAB (Log cfu/g FM^−1^)	5.84
Yeast (Log cfu/g FM^−1^)	5.14
Mold (Log cfu/g FM^−1^)	2.04
Coliform (Log cfu/g FM^−1^)	5.8

^a^DM, dry matter; FM, fresh matter; WSC, water soluble carbohydrate; NDF, neutral detergent fiber; ADF, acid detergent fiber; LAB, lactic acid bacteria.

The count of epiphytic LAB in SSB materials were above 10^5^ cfu/g of FM, which could be attributed to the high WSC content that supports the growth of LAB^24^. Cai reported that the silage can be well fermented when the natural LAB amount reaches over 10^5^ cfu/g of FM in raw materials^12^. The population of initial yeast, mold and coliform ranked from 10^2^ to 10^5^ cfu/g of FM in raw materials. The relatively high mold and coliform counts might indicate the ensiling process of SSB need to be improved by LAB inoculants.

### Effect of different microbial inoculants on chemical composition of SSB silage during ensiling

The dynamics of chemical composition of SSB silages were presented in Table 2. The treatments and storage periods significantly (P < 0.05) affected the DM, WSC, NDF and ADF of SSB silages. But there were no interaction effects between the treatments and storage periods on DM, WSC, NDF and ADF contents. The DM contents in all silages gradually declined with prolonging the ensilage period. In general, the WSC are considered the energy source forcing forage fermentation and thus raising the fermentation quality^25^. In this study, the WSC contents were rapidly decreased in all silages during the first 14 days of ensiling, which were mainly caused by the growth of LAB and undesirable microorganism. The highest WSC contents (145.23 g/kg of DM) were observed in LpE silage after 60 days of ensiling, which could be explained by (a) the low pH and high acidic condition that inhibited the growth of lactic acid bacteria^15^ (b) the release of sugars from the SSB lignocellulosic degradation by cellulase enzyme^26^. As an important factor, the WSC content supports the growth of LAB that produce lactic acid to drop the pH^27^. These results indicated that the SSB forage could be well fermented as good quality silage by proper microbial inoculant, such as LpE presented in this study. With regards to the NDF contents, LpE silages (755.13 g/kg of DM) were lower than other treatments after 60 days of ensiling, which were in agreement with the high WSC contents in LpE silage. The addition of enzymes to inoculant has been reported to degrade fibre and raise the contents of WSC that serve as substrate for LAB fermentation^28^. Many previous studies demonstrated that the plant cell walls of ensiled forages were effectively hydrolyzed by the added cellulase enzyme^29,30^, which were consistent with the findings of our study.

**Table 2:** The dynamics of chemical composition of SSB silage during ensiling.

Items^a^	Treatment^b^	Ensiling time (d)					SEM^c^	Significance		
		3d	7d	14d	30d	60d		T^d^	D	T × D
DM (%, FM)	C	36.76	35.47	34.21	33.77	32.16	0.24	*	**	NS
	CIB	36.20	36.01	35.56	34.70	33.87				
	CIA	37.06	36.52	35.23	34.28	34.07				
	LpE	37.14	36.59	35.79	35.11	34.83				
WSC (g/kg, DM)	C	155.80	146.30	138.30	132.90	129.61	1.32	**	**	NS
	CIB	163.20	158.02	151.20	145.50	138.02				
	CIA	164.10	158.60	149.50	144.10	141.43				
	LpE	162.80	155.70	152.10	150.70	145.23				
NDF (g/kg, DM)	C	785.01	770.89	779.60	773.22	775.53	1.83	**	*	NS
	CIB	783.83	768.83	775.44	760.72	763.72				
	CIA	782.65	769.73	773.34	770.90	765.40				
	LpE	774.66	768.27	764.56	757.32	755.13				
ADF (g/kg, DM)	C	543.52	539.56	542.89	543.21	535.19	3.01	**	*	NS
	CIB	543.90	539.94	528.09	525.29	523.57				
	CIA	546.73	534.63	483.69	510.22	518.93				
	LpE	540.18	530.68	526.76	520.10	519.45				

^a^DM, dry matter; FM, fresh matter; CP, crude protein; WSC, water soluble carbohydrate; NDF, neutral detergent fiber, ADF, acid detergent fiber.

^b^C, Control; CIA, Commercial inoculant A; CIB, Commercial inoculant B; LpE, Isolated lactic acid bacteria inoculant.

^c^SEM, standard error of means.

^d^T, treatment; D, ensilage time; T × D, the interaction between treatment and ensilage time; ^*^P < 0.05; ^**^P < 0.01 NS, not significant.

### Effect of different microbial inoculants on fermentation quality of SSB silages during ensiling

**Figure 1:**
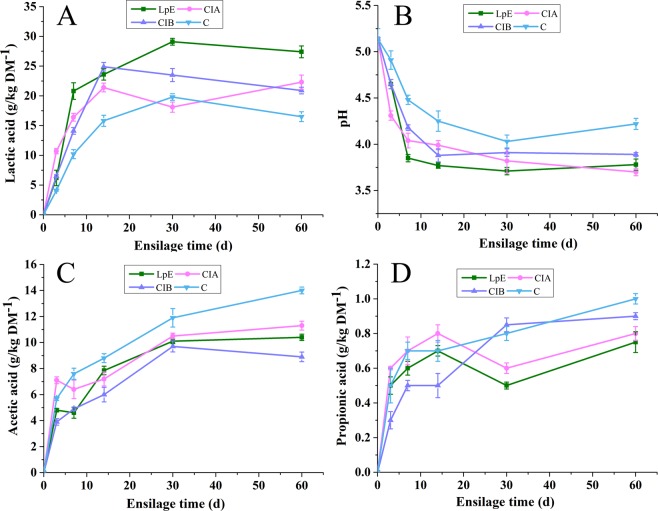
The fermentative changes in pH, lactic acid, acetic acid and butyric acid of SSB silage during the ensiling process. FM, Fresh matter; C, Control; CIA, Commercial inoculant A; CIB, Commercial inoculant B; LpE, Isolated lactic acid bacteria inoculant.

The fermentative changes in lactic acid, pH, acetic acid and propionic acid of SSB silages were presented in Fig. 1 and the calibration curves of organic acids by HPLC are shown in Table 3. With respect to the lactic acid contents, a rapid increase in inoculated silages occurred in the first 7 days of ensiling (Fig. 1A), which indicated that the *L.plantarum* rapidly converted the fermentable substrates into lactic acid, along with the pH rapidly declining^31^. The lactic acid contents in LpE silage were significantly higher (P < 0.05) than corresponding silages and control at the end of ensiling. The pH of silages inoculated with LpE, CIA and CIB inoculant were lower (P < 0.05) than that of control on all ensiling days (Fig. 1B). After 14 days of fermentation, the pH of CIA and CIB remained constant with prolonging the ensilage process. While the LpE inoculated SSB have led to the lowest pH (3.71) among the treatments on day 30, followed by a slight increase. It is generally considered that the addition of homofermentative *L.plantarum* was related to the rapid reduction of pH during the silage fermentation^32^.

**Table 3:** The calibration curves of organic acids (including lactic-, acetic-, propionic-, and butyric acids) by HPLC.

Organic acid	Standard curve^a^	Correlation coefficient(R^2^)
Lactic acid	*Y* = *1.4123X − 1.3115*	0.9999
Acetic acid	*Y* = *1.3941X* + 2.2982	0.9999
Propionic acid	*Y* = *1.3683X − 3.4891*	0.9998
Butyric acid	*Y* = *0.5119X* + *3.1089*	1

^a^Y, organic acid concentrations; X, signal area.

In the present study, all inoculated silages significantly (P < 0.05) reduced the acetic acid contents as compared to control on all ensiling intervals, except for CIA silage on day 3 (Fig. 1C). The high acetic acid contents in control suggests that the natural fermentation of SSB without inoculant is more tend to heterofermentative fermentation, which was in accordance with the report by Shao *et al*.^33^. After 14 days of ensiling, the propionic acid contents in LpE silage were obviously decreased (Fig. 1D), which were likely caused by the low pH and high lactic acid conditions that inhibited the growth of undesirable bacteria. According to Oliveira *et al*. propionic acid was mainly produced by harmful microorganism which decomposed sugars or lactic acid resulting in nutrition loss in the silage^34^. Moreover, all silages were well preserved with no detectable butyric acids (data not shown). These results indicated that the LpE inoculant significantly improved the fermentation quality of SSB, especially enhanced the lactic acid contents, declined the pH and propionic acid contents in the fermentation process.

### Effect of different microbial inoculants on microbial counts of SSB silages during ensiling

**Figure 2:**
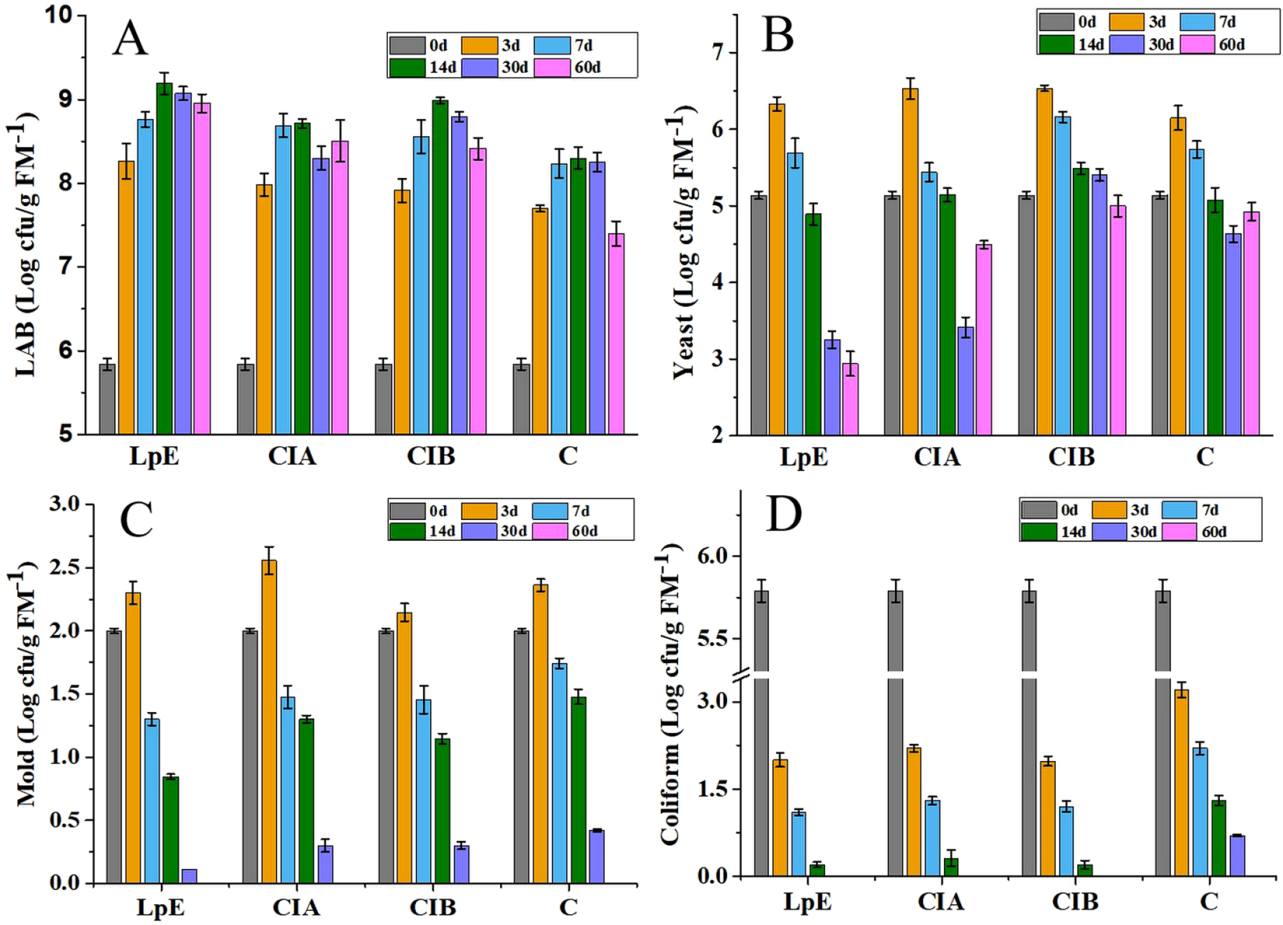
The dynamic changes of microbial counts by plate culture of SSB silage during the ensiling process. Note: The minimum detectable level: there was only one colony growth after the water extracts were directly spread on the corresponding colony count plates without dilutions.

The dynamic changes of microbial counts in inoculated silages and control were showed in Fig. 2. During the first 3 days of fermentation, the number of LAB were significantly changed and rapidly increased to 8.26 ± 0.21, 7.98 ± 0.14, 7.91 ± 0.14 and 7.7 ± 0.04 Log cfu/g FM^−1^ in silage inoculated with LpE, CIA, CIB and C, respectively. Along with the ensilage time, the LAB population in inoculated silages and control were gradually increased and then decreased, with the greatest value appeared at day 14 of ensiling. Nevertheless, the LAB population in silage inoculated with LpE on all ensiling days were higher (P < 0.05) than that in silages inoculated with CIA, CIB and control (Fig. 2A), which might be attributed to the supplementation of fibrolytic enzymes that can degrade the structural carbohydrates of silage into fermentable sugars that supports the growth of LAB^35,36^. In agreement, Colombatto *et al*. demonstrated that the addition of cellulase enzymes and *L.plantarum* could effectively prompt the LAB fermentation and improve the silage quality^37^.

Similarly, the ensiling process also increased the mold and yeast population at the early stage of ensiling (3d), and then declined with prolonging the ensilage process(Fig. 2B,C). After 30 days of ensiling, the mold population decreased to below the detectable level in all silages (P < 0.01). According to Kim *et al*. a high mold and yeast population could reduce the aerobic stability during the feed-out stage and deteriorate the nutritional value of silage^38^. In consideration of acetic acid could decrease the yeasts and fungi and improve the aerobic stability of silage, a few heterofermentative *L.buchneri* were also necessary for silage fermentation^39^. Thus, a functional bacterial inoculant should be involved both of homofermentative *L. plantarum* and heterofermentative *L. buchneri*, so as to inhibit the growth of undesirable bacteria, as well as improve the silage quality^31,40,41^. Although the final yeast population still maintained a relatively high level, LpE treatment showed the fastest rate of reduction (P < 0.05) compared with the CIA, CIB and control treatment, indicating the excellent inhibition effects of LpE inoculant on the growth of yeast and mold. In addition, the coliform population dramatically decreased at the early stage of ensiling process (3 to 14d), and then decreased to below the detectable level after 30 days ensiling in inoculated silages, except for control (Fig. 2D). These results indicated that the LpE inoculant were adequate to dominate the fermentation process of SSB.

### Effect of microbial inoculants on microbial community examined by NGS before and after ensiling of SSB

In order to gain further knowledge regarding the effect of microbial inoculants on ensiling process of SSB, microbial community analysis at the genus level was performed. To the best of our knowledge, this study is the first report of microbial community analysis of SSB silage fermented with microbial inoculant.

Overall, 536,053 quality filtered 16S rRNA sequences were clustered into 545 OTUs based on 97% species similarity. The coverage values of all samples were above 0.99, suggesting that the most of bacterial were detected. The diversity of microbial community in silage samples was presented in Table 4 where a total of 226 to 403 OTUs were detected. Compared to the control silage, the silage fermented with CIA and LpE obtained lower OUTs number. The richness indices Chao, Shannon and Ace of samples showed a similar trend to OTUs, which indicated that the microbial community of inoculated silage had lower richness than that of the control. During the silage fermentation process, the concentrations of organic acids played an important role in the microbial community of silage in that the acidic condition mainly caused by lactic acids and acetic acids could accelerate the decrease in pH, and inhibit the undesirable microbes, and then lower the richness of microbial community in fermented silage^30^. These results were supported by several previous studies that found that the complex microbial community of fresh materials were gradually replaced by LAB added in inoculant resulting in sharply reduce of its richness^42–44^.

**Table 4:** Diversity statistics of microbial community in inoculated silage before and after ensiling.

Silage	Sequence	OTU	Chao	Shannon	Simpson	Ace	Coverage
FM^a^	68257	344	252.07	2.84	0.20	244.36	0.99
C	135954	403	260.73	2.59	0.20	255.56	0.99
CIA	112599	278	214.23	0.88	0.69	202.14	0.99
CIB	106343	374	293.46	1.51	0.48	283.2	0.99
LpE	112900	226	213.49	0.35	0.90	204.07	0.99

^a^FM, Fresh matter; C, Control; CIA, Commercial inoculant A; CIB, Commercial inoculant B; LpE, Isolated lactic acid bacteria inoculant.

**Figure 3:**
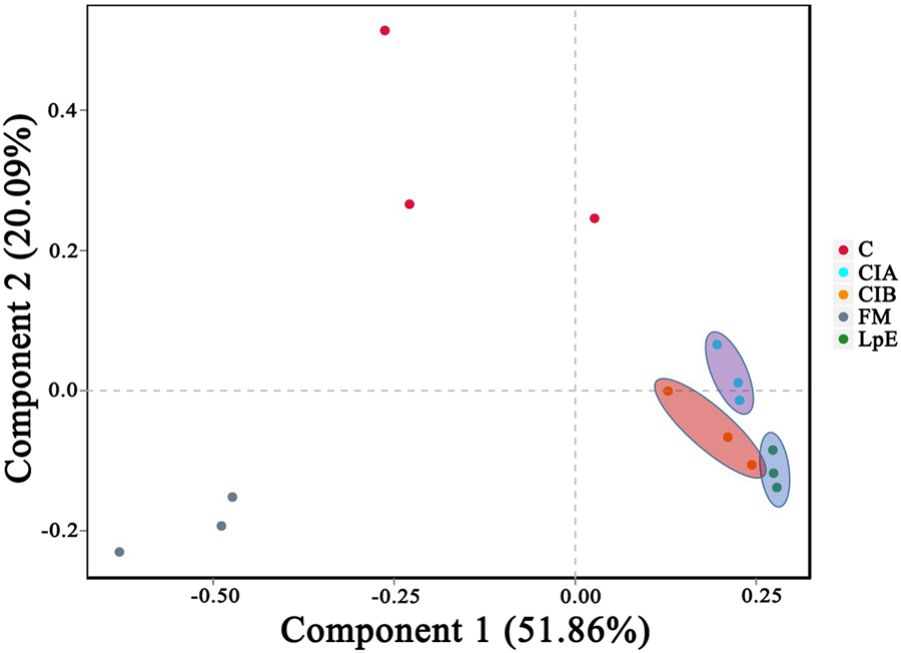
Principal co-ordinates analysis (PCoA) of bacterial communities in SSB before and after ensiling with various microbial inoculants. FM, Fresh matter; C, Control; CIA, Commercial inoculant A; CIB, Commercial inoculant B; LpE, Isolated lactic acid bacteria inoculant.

As shown in Fig. 3, component 1 and component 2 could explain 51.86% and 20.09% of the total variance, respectively. The inoculated silage (LpE, CIA, CIB) were obviously separated from the FM and control (C) samples, indicating that the microbial community differed significantly between treated and untreated SSB silage, and conclude that the excellent inoculant was the main factor underlying limited fermentation quality SSB.

**Figure 4:**
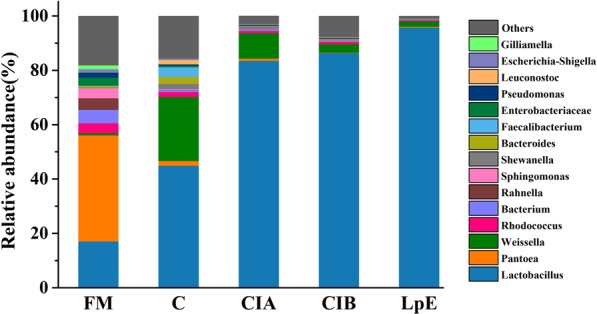
Relative abundance of bacterial at the genus level. FM, Fresh matter; C, Control; CIA, Commercial inoculant A; CIB, Commercial inoculant B; LpE, Isolated lactic acid bacteria inoculant.

The dominant genera in fresh matter of SSB were *Pantoea* (39%), followed by *Lactobacillus* (17.1%), *Bacterium* (0.05%); others accounted 18.2% of total microbial community(Fig. 4), which might be caused by the process of juice extraction that changed the original microbial community structure. The highest relative abundance of *Pantoea* in fresh matter were also detected in soybean materials^15^, but the roles of *Pantoea* in fresh materials were still unclear and need to be further researched. After ensiling, the *Lactobacillus* and *Weissella* were the dominant microbes in all silage samples (LpE, CIA, CIB and Control), but its relative abundance vary with each other. The silage inoculated with LpE were dominated (P < 0.05) by *Lactobacillus* (95.71%), *Weissella* (0.19%); others were below 0.01% of relative abundance. Ogunade *et al*. reported the *Weissella* was obligated heterofermentative bacteria, and it was outcompeted by *Lactobacillus* at the latter stage of fermentation^45^. To date, the strains of *Lactobacillus* genus have been successfully isolated from different crop silages and enhanced the lactic acid fermentation and silage quality as silage additives^24,34,46^. These were supported by our results that *L. plantarum* and *L. buchneri* could achieve good fermentation of SSB ensiling. While the dominant genus in CIA and CIB silage were *Lactobacillus* (83.5% and 86.3%) followed by *Weissella* (0.094% and 0.031%), respectively. Thus, the effect of commercial inoculant (CIA and CIB) on SSB ensiling were similar and it needs to be further improved to achieve high quality fermentation of SSB. The most dominant microbial genus identified of control were *Lactobacillus* (44.92%), *Weissella* (23.55%), others (15.63%), which indicated that the ensiling process of SSB need to be manipulated by using proper microbial inoculants. In addition, the variation of microbial community in silage might be one critical factor leading to differences of silage quality^47^.

## Conclusion

This study comprehensively analyzed the effects of microbial inoculants on the fermentation characteristic and microbial community of SSB silage. The LpE inoculant were more effective in reducing pH value, accumulating lactic acid and achieving good fermentation of SSB silages, indicated that the ensiling of SSB with LpE inoculant could be a feasible way to preserve nutrition and improve fermentation quality. The microbial community analysis represented that the *Lactobacillus* and *Weissella* were the dominant microbes in LpE silage and its relative abundance were 95.71% and 0.19%, respectively. Our study demonstrated that the microbial community analysis will provide a deep insight in SSB fermentation and may be helpful for developing target-based regulation methods to achieve high-quality silage production using SSB.

## Methods

### Chemical composition analysis

Three bags for each treatment were opened after 3, 7, 14, 30 and 60 days of fermentation, respectively. Dry matter (DM) contents were determined following forced air oven at 60 °C for 48 h and ground to pass a 1 mm screen with a Willey mill. Water soluble carbohydrate (WSC) contents were determined using the 3,5-dinitrosalicylic acid (DNS) method^48^. The neutral detergent fiber (NDF) and acid detergent fiber (ADF) contents were determined according to the method descried by Van Soest *et al*.^49^. The contents of lignin, cellulose and hemicellulose in raw SSB were determined using Van Soest method^50^.

### Ensiling of sweet sorghum bagasse

The sweet sorghum (BJ6002) was planted in the experimental field of Institute of Modern Physics, Chinese academy of science (IMP, CAS), Gansu province (latitude 37.93°N, longitude 102.63°E, China), and harvested at maturity stage of growth on 2 October 2018. After harvest, the fresh sweet sorghum were directly juiced using a three-roller mill (ZY-80, Qing shun, Guangdong, China). Then, the sweet sorghum bagasse (SSB) after juice extraction were chopped into 2 cm lengths with manual forage chopper and treated as follows: (1) C, no inoculant control. (2) LpE inoculant, including two LAB strains (*Lactobacillus plantarum* and *Lactobacillus buchneri* with NCBI Accession No. MN022576 and MN022577, respectively) isolated from corn silage and fibrolytic enzyme (200 U/mL) produced in our previous study^51^, and these two isolated LAB strains were mixed at a ratio of 4:1 and applied at 4 × 10^5^ cfu/g FW. (3) CIA inoculant, which were purchased from the BIOVET.Ltd (FermenAider™ Silage Inoculant) and added at rate of 0.038‰ (v/w) FW according to the manufacturer’s protocol, (4) CIB inoculant, which were purchased from the Taiwan Yaxin Biotechnology Co.Ltd (Beijing) and added at rate of 2 × 10^5^ cfu/g FW according to the manufacturer’s protocol. Each inoculant was mixed homogeneous with chopped grass (800 g) and packed manually into a plastic bag, followed by sealing with vacuum preservation system (AJ-320, AODEJU, China). A total of 60 bags (5 ensiling days × 3 repeats × 4 treatments) were made and ensiled at room temperature.

### Fermentation quality for SSB silage

To measure the contents of organic acids and pH, a 10 g sample was mixed with 90 mL of distilled water and homogenized in a blender for 30 min, then filtrated through 0.22 µm membrane filters. The pH was immediately measured with a glass electrode pH meter (PB-10, Sartorius, Germany). Concentrations of organic acids were determined using HPLC (UltiMate 3000, Thermo, USA), which was fitted with a UV detector (VWD-3400RS, 210 nm; column: AT C18 SinoChrom ODS BP; eluent: 0.05 M H_2_PO_3_, 0.5 mL/min) with lactic acid, acetic acid, propionic acid, and butyric acid (Damao, Tianjin, China) as the standards.

### Microbial analysis

#### Microbial counts analysis

The microbial counts were quantified by culture-based method during ensiling. A 10 g fresh sample with 90 mL of sterile saline (0.85% NaCl) was homogenized in a blender for 30 min, and then the water extracts were subjected to serial dilutions ranging from 10^−1^ to 10^−6^ cfu/mL. The counts of lactic acid bacteria (LAB) were measured by plate count on De Man, Rogosa, Sharpe (MRS, Hope, Qindao, China) agar after anaerobic incubation (YQX-II, Yuejin Medical Instrument, China) at 37 °C for 48 h. Molds and yeasts were counted on potato dextrose agar, following incubation at 30 °C for 24 to 72 h, and yeasts were differentiated from molds and other bacteria via colony morphology and appearance^15^. Coliform was measured on Violet Red Bole agar (VRBA, Hope, Qindao, China) incubated at 37 °C for 24 h.

#### Microbial community analysis

The samples of fresh material and silages after 60 days of fermentation were subjected to microbial community analysis. Forage and silage samples (100 g) were added to 500 ml of sterilized phosphate-buffered saline (pH 7.4), and sonicated in an Ultrasonic cleaning bath (VGT-2013QTD, Guangdong GT, China) for 10 min at room temperature. After sonication, the water extracts were subjected to centrifugation at 12000 × g for 15 min, then the bacterial cells were kept at −80 °C. The total microbial DNA were extracted using the TIANamp Bacteria DNA isolation kit (DP302-02, Tiangen, Beijing, China) following the manufacturer’s protocol. Then, the extracted DNA samples were used for amplifying the V3-V4 hypervariable region of 16S rRNA gene with the following universal primer sets: forward, 5′-ACTCCTACGGGAGGCAGCA-3′; reverse, 5′-GGACTACHVGGGTWTCTAAT-3′). The procedures of PCR amplification were followed as described by Ni *et al*.^15^.

### Illumina Miseq sequencing and data analysis

The amplicon libraries were sequenced by paired-end sequencing on an Illumina Miseq platform at the Biomaker Company Co., Ltd. (Beijing, China). For improving the quality of original data, the Trimmomatic (v.0.33) software was used to discard the reads containing barcode or primer errors, and the UCHIME (v.4.2) was used to identify and remove the chimeric sequences. Sequences with low-quality (Q-score ≤ 20) were filtered out, and then the sequences that overlapped more than 50 bp were assembled. After filtering process, the effective tags (at least 200 bp long) were clustered into operational taxonomic units (OTUs) with a threshold of 97% sequence similarity (QIIME v.1.8.0). The OTUs file was used to evaluate the alpha (Mothur v.1.30) and beta diversity (QIIME v.1.8.0) of SSB silage inoculated with various microbial inoculant.

### Statistical analysis

The statistical analysis were carried out using the SPSS 20.0 (SPSS Inc., Chicago, IL, USA) and Origin 9.0 (Origin Lab Corp., Northampton, MA, USA). The effects of treatments, storage periods and treatments by storage periods interaction were analyzed by two-way ANOVA by using the GLM procedure of the SPSS 20.0. Tukey’s test was employed for different sample means and the significance was declared at P < 0.05.
